# Hepatocellular carcinoma in Gaucher disease: an international case series

**DOI:** 10.1007/s10545-018-0142-y

**Published:** 2018-02-08

**Authors:** Martine Regenboog, Laura van Dussen, Joanne Verheij, Neal J. Weinreb, David Santosa, Stephan vom Dahl, Dieter Häussinger, Meike N. Müller, Ali Canbay, Miriam Rigoldi, Alberto Piperno, Tama Dinur, Ari Zimran, Pramod K. Mistry, Karima Yousfi Salah, Nadia Belmatoug, David J. Kuter, Carla E. M. Hollak

**Affiliations:** 1 Department of Internal Medicine, division of Endocrinology & Metabolism, Academic Medical Center, Meibergdreef 9, 1105 AZ Amsterdam, The Netherlands; 2 Department of Pathology, Academic Medical Center, Amsterdam, The Netherlands; 3 Department of Human Genetics and Medicine, University of Miami, Miller School of Medicine, Miller, FL USA; 4 Department of Gastroenterology, Hepatology and Infectious Diseases, Heinrich-Heine University, Düsseldorf, Germany; 5 Department of Gastroenterology, Hepatology and Infectious Diseases, Otto-von-Guericke University, Magdeburg, Germany; 6 Medical Genetics, University of Milano-Bicocca and ASST-Monza, S. Gerardo Hospital, Monza, Italy; 7 Gaucher Clinic, Shaare Zedek Medical Center, Jerusalem, Israel; 8 Department of Internal Medicine, Yale University School of Medicine, New Haven, USA; 9 Department of Internal Medicine, Referral Center for Lysosomal Diseases, University Hospital Paris Nord Val de Seine, site Beaujon, Clichy, France; 10 Department of Hematology, Massachusetts General Hospital, Harvard Medical School, Boston, MA USA

**Keywords:** Gaucher disease, Hepatocellular carcinoma, Risk factors, Liver fibrosis, Malignancy, Case series

## Abstract

Gaucher disease (GD) is associated with an increased risk for malignancies. Next to hematological malignancies, the development of solid tumors in several organs has been described. The liver is one of the major storage sites involved in GD pathogenesis, and is also affected by liver-specific complications. In this case series, we describe 16 GD type 1 (GD1) patients from eight different referral centers around the world who developed hepatocellular carcinoma (HCC). Potential factors contributing to the increased HCC risk in GD patients are studied. Eleven patients had undergone a splenectomy in the past. Liver cirrhosis, one of the main risk factors for the development of HCC, was present in nine out of 14 patients for whom data was available. Three out of seven examined patients showed a transferrin saturation > 45%. In these three patients the presence of iron overload after histopathological examination of the liver was shown. Chronic hepatitis C infection was present in three of 14 examined cases. We summarized all findings and made a comparison to the literature. We recommend that GD patients, especially those with prior splenectomy or iron overload, be evaluated for signs of liver fibrosis and if found to be monitored for HCC development.

## Introduction

The lysosomal storage disorder Gaucher disease (GD) results from a deficiency of the enzyme glucocerebrosidase (acid β-glucocerebrosidase, EC 3.2.1.45) which is involved in the breakdown of the glycosphingolipid glucocerebroside (Brady et al 1965). In this autosomal recessively inherited disease, mutations in the gene encoding glucocerebrosidase (GBA1-gene) are the cause of deficient enzyme activity and result in accumulation of glucocerebroside in tissue macrophages. Major storage sites include spleen, liver, and bone marrow leading to a heterogeneous clinical picture with symptoms of hepatosplenomegaly, cytopenia, and bone disease. GD is phenotypically classified into three variants; type 1 is the most common, type 2 and 3 are the rare neuronopathic (acute and subacute respectively) forms (Grabowski 2008). Therapy options include enzyme replacement therapy (ERT) and substrate reduction therapy, which have proven to be highly effective in reversing clinical signs and symptoms (Barton et al 1991; Weinreb et al 2013; Cox et al 2015). Before the discovery of ERT, splenectomy was frequently performed in patients suffering from symptoms of severe splenomegaly. It has been hypothesized that after splenectomy patients are at an increased risk of liver complications, i.e., fibrosis and cirrhosis, and more extensive bone involvement (Fleshner et al 1991; Lachmann et al 2000; Bohte et al 2013).

Over the years, it has become apparent that GD is associated with an increased risk of malignancies. In additoin to hematological malignancies, solid tumors in several organs have been described (Shiran et al 1993; Zimran et al 2005; de Fost et al 2006; Taddei et al 2009; Lo et al 2010; Weinreb and Lee 2013; Arends et al 2013). A previously reported complication in GD is the occurrence of hepatocellular carcinoma (HCC) (Breiden-Langen et al 1991; Erjavec et al 1999; Xu et al 2005).

HCC in the general population is mainly diagnosed in patients affected by a chronic liver disease, such as hepatitis B or C infection. The presence of cirrhosis is a main risk factor for the development of HCC (Forner et al 2012; El-Serag 2011). However, HCC can also occur in a non-cirrhotic liver (Schütte et al 2014; Van Meer et al 2016). Other risk factors include non-alcoholic fatty liver disease (NAFLD), hereditary haemochromatosis (HH) diabetes mellitus (DM), alcohol use, smoking, and obesity (Degasperi and Colombo 2016; Kowdley 2004; El-Serag et al 2004; Marrero et al 2005). Surveillance for HCC by biannual ultrasound examination is recommended for patients with a cirrhotic liver, irrespective of its etiology, and high-risk hepatitis B/C carriers (Diáz-González and Forner 2016).

To date it is unknown which GD patients are especially at risk for developing HCC. Advanced liver involvement after splenectomy with subsequent fibrosis and cirrhosis, as stated above, has been suggested as a potential mechanism for the increased risk (de Fost et al 2006). It is of interest to ascertain the factors, which contribute to the development of HCC in GD in order to be able to implement a rational screening protocol to detect these cancers at an early stage. With this international collaborative study we aim to summarize clinical and pathological findings of GD patients who developed a HCC in the past. We discuss the possible factors contributing to the development of this hepatic malignancy in relation to GD, compare our findings to existing literature on this topic and suggest a follow-up strategy for screening.

## Methods

We retrospectively studied medical files of 16 type 1 GD patients with a confirmed HCC diagnosis from Gaucher referral clinics in the Netherlands, the United States of America, Germany, Italy, Israel, and France. Some of the cases have been previously described in the literature. All cases are described in terms of clinical, laboratory, and imaging findings. Parameters recorded for every patient include gender, age at GD diagnosis, genotype, spleen status, GD treatment, age at start of GD treatment, presence of bone disease, age at HCC diagnosis, laboratory findings at time of HCC diagnosis (iron status, alfa-foetoprotein, liver enzymes), known risk factors of HCC (hepatitis B/C, alcohol abuse, HFE-mutation, history of blood transfusions), imaging findings, therapy, complications, outcome, and pathology findings.

## Results

### Clinical data

A summary of patient characteristics is shown in Table 1. In all patients a diagnosis of Gaucher disease was confirmed based upon deficient activity of GBA and/or GBA1 mutation analysis. All patients had type 1 disease. A total number of 16 patients have been included, of whom ten patients (63%) were male. Eleven patients were splenectomized in the past. Mean age at which GD diagnosis was made was 26 years (range 3-60 years). The majority of patients (*n* = 14) were treated with ERT. The mean time between GD diagnosis and start of ERT was 20 years (range 0-41 years). Mean age at which HCC diagnosis was confirmed was 58 years (range 28-76 years). Therapeutic options for HCC, which have been applied in the studied cases include surgical resection (*n* = 6), liver transplantation (*n* = 2), percutaneous ablation (*n* = 3), transarterial chemoembolization (TACE; n = 6), immunotherapy (n = 1), chemotherapy (n = 1), radiotherapy (n = 1), and brachytherapy (n = 1) with some patients being treated with multiple treatment modalities. Three patients in this series are still alive after liver transplantation (case no. 12), resection of the liver tumor (case no. 14) or brachytherapy (case no. 16). All but one of the remaining 13 patients died as a consequence of the hepatocellular carcinoma or its complications. In one case (no. 5) another type of malignancy (gastro-esophageal, GE) was reported as final cause of death. This patient suffered from multiple malignancies beside the HCC and GE carcinoma.

**Table 1:** Characteristics of GD1 patients with a diagnosis of HCC

No.	Country	Gender	Age at GD diagnosis	Genotype	Sx (age at Sx)	Bone disease	GD therapy (age at start)	Age at HCC diagnosis	Course and comorbidities or complications	Outcome (age at death)
1*	NL	male	36	N370S/I260T	Yes (53)	yes	Alglucerase (61)	62	Metastatic lesions peritoneum, omentum	Died (63)
2*	NL	male	33	N370S/N370S	Yes (33)	yes	Alglucerase, imiglucerase (44)	63	Resection of tumor, ablation, liver transplantation Recurrence of HCC	Died (69)
3*	NL	female	7	N370S/L324P	Yes (13)	yes	Alglucerase, imiglucerase (37)	55	Resection of tumor, TACE-procedure Respiratory insufficiency, kidney failure	Died (55)
4*	NL	female	18	R463C/?	Yes (18)	no	Alglucerase, imiglucerase (34), miglustat	39	Vena porta thrombosis	Died (39)
5	USA	male	10	N370S/L444P	Yes (36)	yes	Alglucerase, imiglucerase, velaglucerase (51)	68	Resection of tumor Multiple malignancies: bladder- and prostate carcinoma, gastro-oesophageal carcinoma	Died (75)
6	GER	male	50	*Missing*	No	yes	Imiglucerase (50)	61	TACE-procedure (3×) Recurrence of HCC, variceal bleeding	Died (62)
7	GER	female	28	N370S/?	Yes (28)	yes	Alglucerase, imiglucerase, velaglucerase (51)	68	Resection of tumor, TACE-procedure Recurrence of HCC, metastatic bone lesion	Died (71)
8	GER	male	8	*missing*	Yes (8)	no	Imiglucerase (8) eliglustat (trial)	28	Resection Recurrence of HCC	Died (31)
9	IT	male	41	N370S/F213I	Yes (41)	yes	Not treated	60	Ablation (3×), TACE-procedure Recurrence of HCC, metastasis	Died (65)
10*	ISR	female	5	N370S/84GG	Yes (16)	yes	Alglucerase (16)	37	Lung metastasis Ablation, xeloda	Died (unknown)
11	ISR	male	60	N370S/N370S	No	no	Not treated	*missing*		Died (unknown
12*	USA	female	3	N370S/84GG	Yes (8)	yes	Alglucerase (37)	47	TACE, Liver transplantation, breast cancer	Alive
13	FR	male	57	N370S/L324P	No	yes	Imiglucerase, velaglucerase (74)	76	Venous thrombosis lower limbs, anemia, portal hypertension TACE-procedure	Died (unknown)
14	FR	female	9	N370S/ IVS2 G(+1)-T	Yes (18)	yes	Alglucerase, imiglucerase, velaglucerase (48)	73	Resection of tumor	Alive
15	USA	male	18	84GG/1226G	no	yes	Alglucerase, imiglucerase (53)	72	SBRT, nivolomab, TACE Progressive HCC, no response to therapy	Died (74)
16	GER	male	37	N370S/?	no	yes	Imiglucerase (37)	58	CBT	Alive

Abbreviations: NL, the Netherlands; USA, United States of America; GER, Germany; IT, Italy; ISR, Israel; FR, France. GD, Gaucher disease; HCC, hepatocellular carcinoma; Sx, splenectomy; TACE, transarterial chemo-embolisation; SBRT, stereotactic body radiotherapy; CBT, CT-guided brachytherapy

*Case previously described in literature

Cases 1, 2 , 3, and 4 in Arends et al (2013), case 1 also in case report by Erjavec et al (1999), cases 1 and 4 in de Fost et al (2006), case 10 Zimran et al (2005), case 12 case report by Xu et al (2005)

### Laboratory findings and predisposing factors for HCC

In Table 2 the laboratory data at time of HCC diagnosis is provided. Alfa-foetoprotein (αFP) levels show mild to extreme elevations in all but one case for whom data was available (*n* = 13). Information about hepatitis infection was available in 14 patients; this data was missing in two patients. Three of 14 patients were diagnosed with chronic hepatitis C infection (case no. 3, 9, and 10), and all showed elevated liver enzymes at time of HCC diagnosis. In nine hepatitis negative patients liver enzymes also showed mild elevations. Analysis of HFE-gene mutations was performed in six patients; one was homozygous for the C282Y mutation, one for C282Y/H63D mutations, and one heterozygous for H63D. A history of blood transfusion was recorded for six out of the nine patients for whom data was available. Ferritin levels were reported for 11 patients and increased in eight cases. Three out of eight patients, in whom transferrin saturation (Tsat) was analyzed, showed increased Tsat levels (>45%). Iron overload in these cases was confirmed on histopathological examination. These patients are among the six patients with a known history of blood transfusions.

**Table 2:** Laboratory data and presence of predisposing factors for HCC of GD patients at time of HCC diagnosis

No.	Sx	AFP (μg/L)	ALAT (U/L)	ASAT (U/L)	AF (U/L)	γGT (U/L)	Albumin (g/L)	Ferritin (μg/L)	Transferrin saturation (%)	Hepatitis B/C serology	HFE-mutation	Blood transfusion	Alcohol abuse*	Presence of fibrosis / cirrhosis	Iron staining
1	Yes	83,628	23	61	117	97	41	995	31	negative	–	–	No	- / -	Np
2	Yes	18	53	53	86	–	38	3334	65	negative	H63D/wt	Yes	No	+ / +	+
3	Yes	248	68	93	137	189	37	3754	91	Hep.C +	wt/wt	Yes	No	+ / +	+
4	Yes	16,281	60	416	341	372	29	441	15	negative	–	–	No	unknown	Np
5	Yes	10.5	24	–	–	–	–	124	–	negative	–	No	No	- / -	–
6	No	6.47 (ref <6.2 ng/ml)	46	43	97	154	42	23	–	negative	–	Yes	No	+ / +	Np
7	Yes	26.1 ng/ml (ref <8.1	16	43	120	62	43	–	–	negative	–	–	No	- / -	Np
8	Yes	41 ng/ml	31	35	92	58	44	84	24	negative	–	–	No	+ / +	Np
9	Yes	44.6	91	69	211	106	44	4470	46	Hep. C+	Wt/wt	Yes	–	- / -	+
10	Yes	29,252	38	435	593	1228	2.5?	–	–	Hep. C+	–	Yes	–	? / +	Np
11	No	–	16	20	16	21	4.3?	31	–	–	C282Y/C282Y	–	–	unknown	Np
12	Yes	161	47	102	189	786	24	–	–	Hep. B +	–	–	No	+ / +	Np
13	No	–	105	80	204	440	41	463	25	Negative	C282Y/H63D	Yes	–	? / +	Np
14	Yes	–	16	25	87	33	40	389	–	Negative	–	No	–	? / +	Np
15	No	68	38	47	682	–	35	1059	7	Negative	Wt/wt	No	No	- / - **	–
16	No	6	43	29	68	217	41	–	–	–	–	–	No	? / +	Np

Abbreviations: Np, not performed; Sx, splenectomy

*Alcohol abuse is defined as prolonged intake of 40-60 g of alcohol/day (standard drink containing 13.7 g) (El-Serag 2011) or a known history of alcohol abuse

**Histopathological examination only revealed tumorous tissue, no normal liver tissue. Although not confirmed, the presence of cirrhosis in this case is suspected

### Histopathological findings

**Fig. 1:**
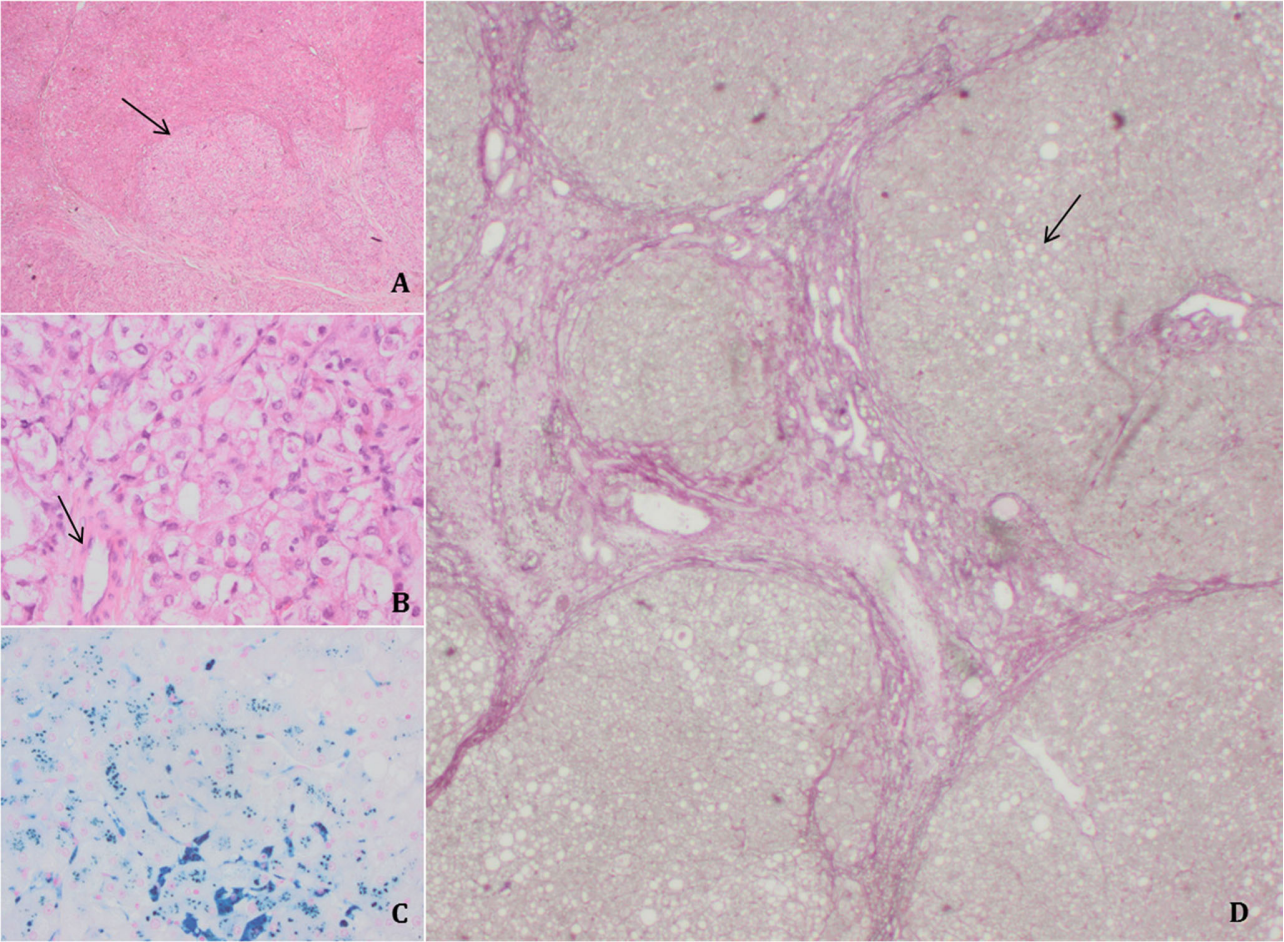
Histology findings of liver tissue of case no. 2 **a** Clear demarquation of HCC lesion and liver parenchyma (arrow); **b** Higher magnification with clear atypia and solitary artery (arrow); **c** Prussian blue staining, indicating the presence of iron in hepatocytes and Kupffer cells in blue; **d** Surrounding liver characterized by cirrhosis with nodular architecture and presence of fibrous bands (red). There is also the component of steatosis (arrow)

Pathology information was available for 14 cases (see Table 2). In nine cases the presence of liver cirrhosis was reported. In one case (no.15) only tumorous tissue was found on histopathological examination. The presence of cirrhosis in surrounding liver tissue is suspected but not confirmed. The remaining four patients did not show evidence for fibrosis or cirrhosis. Iron staining of liver tissue was described in five cases, of which three were found positive (case no. 2, 3, and 9). Iron loading was present in macrophages as well as in hepatocytes up to grade 3. In these three cases, Tsat-values all exceed 45% as described in the previous paragraph. Cases 2 and 3 were also found to have a cirrhotic liver, whereas case no. 9 did not show evidence of fibrosis or cirrhosis. An example of histological findings is shown in Fig. 1.

### Imaging findings

Data on imaging findings is missing in three cases. In the remaining 13 cases imaging characteristics of the lesions have been described in ultrasound (US) in five cases, magnetic resonance imaging (MRI) in six cases, and computed tomography (CT) in 11 cases. The characteristics of the carcinomas appearing on the available imaging modalities vary. In five cases in which contrast imaging was performed, all lesions showed arterial enhancement. In three of these cases the typical wash-out of contrast agent was also described. The presence of multiple malignant lesions in the liver, i.e., multifocal hepatocellular carcinoma, has been described in nine cases. An additional imaging modality used to assess the degree of liver stiffness is transient elastography (TE; Fibroscan©). In three patients (case no. 2, 6, and 7) this method was used, and in all three patients increased liver stiffness values were reported, suggesting the presence of liver fibrosis. Based on validation in non-GD patients and observation in a small cohort of GD (Guo et al 2017, Bohte et al 2013), it is likely that elastography in GD represents fibrotic changes, although lysosomal accumulation by itself could contribute to this.

## Discussion

This study describes a worldwide case series of 16 GD patients who were diagnosed with HCC. Two patients in this study have been described in detail previously (case no. 1 (Erjavec et al 1999) and case no. 12 (Xu et al 2005). Two other cases of HCC reported in the literature were found that have not been included in this series. In 1982, Lee described a 67-year old GD patient who died from HCC (Lee 1982) and another case report was published in which a 48-year old GD patient with HCC in a cirrhotic liver was discussed. This patient also suffered from hepatitis B (Breiden-Langen et al 1991). All cases previously published, were GD patients who had undergone splenectomy in the past. A summary of published cases was provided by Arends et al (2013) and six of the cases described were also included in this series.

In our current study, we have shown that HCC also occurs in GD patients with an intact spleen (*n* = 5). Liver cirrhosis or fibrosis was present in nine patients. Of importance, four cases were described in which HCC developed in a non-cirrhotic liver. One patient (no. 15) had no pathologically confirmed cirrhosis (biopsy retrieved only malignant tissue and no surrounding nonmalignant tissue) but had presented 20 years before the HCC diagnosis with liver failure and had been assumed by his physician to have had cirrhosis. Three patients were shown to have iron overload in the liver, one of those (no. 9) did not show any sign of fibrosis or cirrhosis, whereas the other two iron-overloaded cases had concomitant cirrhosis. The concurrence of a previous hepatitis B infection in one patient and chronic hepatitis C in three other cases points to this as another important risk factor for HCC. In one case the presence of homozygosity for the C282Y mutation in the HFE-gene indicates that this patient possibly suffers from hereditary haemochromatosis as well as GD. However, detailed information regarding this case was missing.

In a liver affected by GD, replacement of normal liver tissue by pathological storage cells can induce the development of a spectrum of abnormalities (James et al 1981). In the pre-ERT era, splenectomy was frequently performed in GD patients. It is thought that after removal of the spleen, advanced hepatic involvement in the GD storage process is a main risk factor for liver-related complications (de Fost et al 2006). It has been shown that splenectomized GD patients do show significantly higher liver stiffness values as compared to GD patients with an intact spleen (Bohte et al 2013). The risk of liver-related complications in this population is associated with the presence of liver fibrosis (James et al 1981; Lachmann et al 2000). In addition, it should be noted that a population of Gaucher cells associated with fibrous septa in the liver does not disappear in response to ERT, indicating the presence of residual storage which is insensitive to the administered enzyme (Lachmann et al 2000; Perel et al 2002).

The mechanisms responsible for the increased risk for malignancies in GD are not fully understood and several factors have been implicated to play a role. The main factors related to the primary genetic defect in GD include immune dysregulation, chronic macrophage activation inducing elevated release of several pro- and anti-inflammatory cytokines, cellular dysfunction due to the accumulation of glucocerebroside and glucosylsphingosine (Arends et al 2013; Mistry et al 2013). The etiology of hepatic carcinogenesis in GD patients is likely to be a result of the abovementioned factors together with the presence of other, HCC-specific, risk-factors and coexistent conditions.

In 80-90% of the patients with HCC worldwide, liver cirrhosis is the underlying cause (Fattovich et al 2004). In nine cases in the current study, the presence of fibrosis and/or cirrhosis was confirmed; in one it was highly suspected. Advanced hepatic involvement of GD after splenectomy could explain the progression of liver disease. However, three patients had an intact spleen but did develop liver cirrhosis. One of those (no. 13) was recorded to have the C282Y/H63D mutation of the HFE-gene, indicating that iron overload as a consequence of hereditary hemochromatosis might contribute to the development of HCC. Two others with intact spleen and liver cirrhosis (case no. 6 and 16) were not reported to have any clear other risk factors, although some data is missing regarding these cases. As liver fibrosis is the preceding histological hallmark of liver cirrhosis, detecting fibrosis is an important step in defining patients at risk for cirrhosis and subsequent HCC development in GD.

An important finding of the current study is that in four GD patients HCC developed in a liver with no signs of fibrosis or cirrhosis (no. 1, 5, 7, and 9). All were splenectomized in the past. The time in between GD diagnosis and start of ERT treatment is 25, 41, and 23 years for cases 1, 5, and 7 respectively. This long untreated state of those patients could potentially contribute to the increasing pathological Gaucher cell burden and subsequent general risk of malignancy. Case 9 was not treated with ERT. Aside from GD, he also suffered from hepatitis C and did develop severe iron overload. Taken together, these factors could all contribute to the occurrence of HCC. Three of four non-cirrhotic GD patients who developed HCC were treated with alglucerase in the past. Alglucerase was the first macrophage-targeted enzyme preparation available (Barton et al 1991). This enzyme preparation was produced by purifying glucocerebrosidase from human placental tissue, and as a consequence, contains human choriogonadotropin (hCG). HCG can be produced by liver tumors, and as such, hCG may have been contributory to the development of HCC (Erjavec et al 1999; Nakanuma et al 1986).

Extremely elevated serum ferritin levels in GD patients despite adequate treatment with ERT or SRT are associated with increased iron storage (Regenboog et al 2017). This finding is confirmed in the current study, in which three patients with serum ferritin levels exceeding 3000 μg/l and Tsat >45% did show evidence of iron loading on histology examination of liver tissue. When available, quantitative magnetic resonance imaging (MRI) of iron might serve as an additional tool in diagnosing and monitoring iron overload (Wood 2014). MRI iron measurements were not available for the cases described. GD itself could be the explanation for a distorted iron metabolism. However, the co-existence of pathological mutations in the HFE-gene should not be missed. As hepatic iron overload can cause toxicity and is associated with carcinogenesis (Kowdley 2004), it is considered a potential contributing factor to the HCC-risk in GD patients.

A limitation of the current study is the fact that for some investigated risk factors, data were not complete. In addition, risk factors for HCC development in general, for example smoking status, the presence of diabetes mellitus or obesity were not recorded for most patients and therefore not included. Indeed, metabolic syndrome is probably more frequent in GD. However, in the Dutch cohort, none of the patients who developed HCC had signs of metabolic syndrome. This does not exclude the possibility that these factors could have contributed to the malignant transformation of liver tissue in our studied cohort.

In current practice, we strongly advise careful surveillance of GD patients at risk for HCC. Early detection of possible malignant lesions might prevent HCC-related mortality in this population. It is important to define the GD patients who are at risk for HCC. Given the importance of fibrosis in identifying patients at risk for HCC, practical recommendation could be to investigate all GD patients for the presence of liver fibrosis by transient elastography (TE; Fibroscan ©). Based on clinical expertise, literature, and the current case series subsequent close follow-up for the development of HCC would be limited to those GD patients for whom 1 or more of the following risk factors is present:

- Splenectomized patients
- Presence of liver fibrosis/cirrhosis
- Persistent hyperferritinemia (> 2 times upper limit of normal) despite adequate GD-specific treatment in combination with transferrin saturation > 45%
- Chronic hepatitis B/C carriers

According to the guidelines for HCC-surveillance of the European Association for the Study of the Liver, a 6-month interval for surveillance is preferable (*EASL-EORTC clinical practice guidelines*^2012^). First-choice testing for HCC surveillance is ultrasound (US) examination. The use of alpha-foetoprotein (AFP) as a serological marker for HCC together with US is not recommended. Since the incidence of HCC is probably very low, and in Western Countries with effective treatment available perhaps even lower, a review of prospectively detected cases by screening should be performed to evaluate the (cost-) effectiveness of the proposed strategy.

In summary, HCC in GD patients is shown to occur in non-cirrhotic as well as cirrhotic livers, irrespective of splenectomy status, although splenectomy itself seems to be a risk factor for fibrosis/cirrhosis. Other contributing factors, such as iron overload and the presence of hepatitis, have been detected. This cohort of patients is characterized by the presence of severe and longstanding disease. It should be clear that most of the patients are diagnosed with GD prior to the discovery and implementation of ERT. The future will show whether timely diagnosis and start of therapy for GD also decreases the risk of malignancies.
